# A framework for evaluating health system surveillance sensitivity to support public health decision-making for malaria elimination: a case study from Indonesia

**DOI:** 10.1186/s12879-022-07581-2

**Published:** 2022-07-15

**Authors:** Riris Andono Ahmad, Luca Nelli, Henry Surendra, Risalia Reni Arisanti, Dyah Ayu Shinta Lesmanawati, Isabel Byrne, Elin Dumont, Chris Drakeley, Gillian Stresman, Lindsey Wu

**Affiliations:** 1 Centre for Tropical Medicine, Faculty of Medicine, Public Health and Nursing, Universitas Gadjah Mada, Yogyakarta, Indonesia; 2 Department of Biostatistics, Epidemiology and Population Health, Faculty of Medicine, Public Health and Nursing, Universitas Gadjah Mada, Yogyakarta, Indonesia; 3 Institute of Biodiversity, Animal Health and Comparative Medicine, University of Glasgow, Glasgow, G11 7RD UK; 4 Department of Infection Biology, London School of Hygiene and Tropical Medicine, London, WC1E 7HT UK; 5 Eijkman-Oxford Clinical Research Unit, Jakarta, Indonesia

**Keywords:** Care seeking, Malaria elimination, Freedom from infection, Global health, Public health, Decision-making, Surveillance sensitivity

## Abstract

**Background:**

The effectiveness of a surveillance system to detect infections in the population is paramount when confirming elimination. Estimating the sensitivity of a surveillance system requires identifying key steps in the care-seeking cascade, from initial infection to confirmed diagnosis, and quantifying the probability of appropriate action at each stage. Using malaria as an example, a framework was developed to estimate the sensitivity of key components of the malaria surveillance cascade.

**Methods:**

Parameters to quantify the sensitivity of the surveillance system were derived from monthly malaria case data over a period of 36 months and semi-quantitative surveys in 46 health facilities on Java Island, Indonesia. Parameters were informed by the collected empirical data and estimated by modelling the flow of an infected individual through the system using a Bayesian framework. A model-driven health system survey was designed to collect empirical data to inform parameter estimates in the surveillance cascade.

**Results:**

Heterogeneity across health facilities was observed in the estimated probability of care-seeking (range = 0.01–0.21, mean ± sd = 0.09 ± 0.05) and testing for malaria (range = 0.00–1.00, mean ± sd = 0.16 ± 0.29). Care-seeking was higher at facilities regularly providing antimalarial drugs (Odds Ratio [OR] = 2.98, 95% Credible Intervals [CI]: 1.54–3.16). Predictably, the availability of functioning microscopy equipment was associated with increased odds of being tested for malaria (OR = 7.33, 95% CI = 20.61).

**Conclusions:**

The methods for estimating facility-level malaria surveillance sensitivity presented here can help provide a benchmark for what constitutes a strong system. The proposed approach also enables programs to identify components of the health system that can be improved to strengthen surveillance and support public-health decision-making.

## Background

As part of the Global Technical Strategy for Malaria endorsed by the World Health Organization (WHO) in 2015, the malaria community has set targets to achieve elimination in over 35 countries by 2030 [1]. Through the E-2025 Initiative launched in 2021, the WHO is supporting 25 countries with the potential to eliminate malaria in the next five years [2]. The rate of progress towards elimination depends not only on biological and environmental factors, but on the strength of the national health system. In endemic settings, robust surveillance systems are needed to ensure that all cases are detected, properly managed by healthcare providers (both public and private) and reported to national malaria registries.

In settings striving to achieve and confirm malaria elimination, having an effective surveillance system is paramount. The WHO malaria elimination certification process requires countries to accurately document no indigenous cases for at least three consecutive years and demonstrate a fully functional surveillance and response system that can adequately prevent re-establishment of indigenous transmission [3]. The functionality of a system is typically assessed using programme audits to assess a range of capacities, including protocols in place to quickly respond to any case detected and national reference laboratories to ensure high-quality diagnostic capacity. Although the audits are able to provide context to the structures in place to support malaria elimination, they do not directly quantify how effective a system is at detecting clinical cases or asymptomatic infections if they are present in a population.

The “Freedom from Infection” (FFI) framework, previously used in veterinary epidemiology, was designed to approach this issue quantitatively. One practical element of the FFI methodology includes an approach to directly estimate the Surveillance System Sensitivity (SSe), or the estimated probability that infected individuals, both symptomatic and asymptomatic, will be detected by the surveillance system [4]. Here, we present a more refined approach to quantify the malaria SSe implemented within a Bayesian framework of statistical inference, using Indonesia as a case study.

Indonesia is one of nine malaria endemic countries in South East Asia, accounting for 21% of the regional cases and 16% of the malaria deaths [5]. In the last decade, accelerated progress towards elimination has led to a 50% reduction in malaria cases and a 66% decline in malaria deaths, putting them on track for their elimination target of 2030. More than half (266 out of 514) of the districts were declared malaria free by 2017, and 93 districts have transitioned from high or moderate transmission to moderate or low transmission [6–8].

Malaria elimination policy in Indonesia has been driven at the subnational level, including targets for mandatory laboratory confirmation of all malaria cases and improved surveillance and reporting in endemic areas [8]. Subnational verification of malaria elimination is an option for large countries or geographically isolated territories, such as islands, where interruption of local transmission in certain parts of the country is feasible or already achieved. This provides an important internal milestone on the path to national certification [4].

The aim of the study was to provide a framework to quantify the sensitivity of the malaria surveillance system in Kulon Progo and Magelang, two districts at different stages of malaria elimination, on Java Island, Indonesia. This is a region with the highest number of malaria cases in Java Island due to an environment favourable for vector breeding [9]. Using survey data to assess the capacity of the health system to support malaria, combined with monthly data from the routine passive case detection system from 2017 to 2019, we developed a model framework to assess the overall sensitivity of the health facility level surveillance system to detect malaria in the community. The health systems survey used for data collection in this study was also designed to align with national and WHO malaria elimination audits used to assess health systems surveillance capacity. This model and its outputs can be embedded as a core structure of future efforts to improve surveillance systems and when applying the FFI concepts to support subnational progress towards malaria elimination targets in these districts.

## Methods

### Study area

Seventy percent of the Indonesian population resides in Java Island, and foci of persistent endemic malaria still remain [9]. We have chosen to focus on Magelang and Kulon Progo districts as examples of areas in two different stages of malaria elimination and historical epidemiological trends. Magelang District in the Central Java Province was certified malaria-free in 2014, though with an epidemic in 2015, there is a need for continued surveillance to prevent reintroduction. Kulon Progo District in Yogyakarta Province is nearing elimination [10], with an increasing number of subdistricts becoming malaria-free since 2000 [11]. Central Java Province has a malaria elimination target of 2023, and malaria cases have shown a downward trend between 2014 and 2016 [12].

### Data collection

**Fig. 1:**
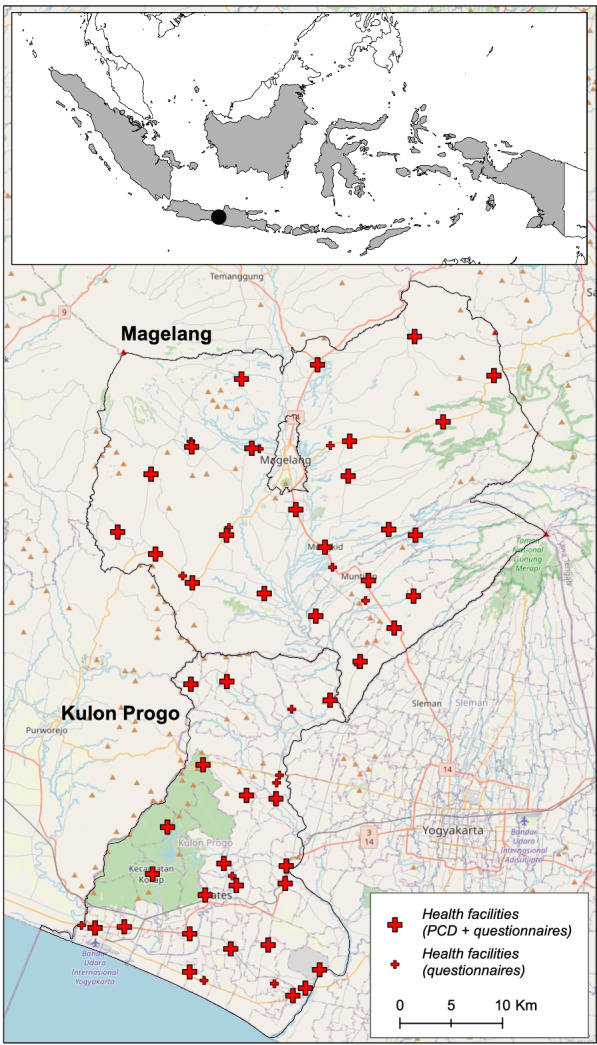
Magelang and Kulon Progo Districts in Indonesia, and location of health facilities. Background images source: OpenStreetMap

Data collection took place between December 2019 and January 2020 using a health systems questionnaire completed by the clinician typically responsible for diagnosing and managing malaria cases. The health systems questionnaire, developed by malaria experts at the Universitas Gadjah Mada (UGM), and the London School of Hygiene and Tropical Medicine (LSHTM), was conducted via in person interviews with each health facility’s malaria programme manager, at a subset of facilities in Kulon Progo and Magelang (Fig. 1). In Kulon Progo, a total of 30 health facilities were interviewed, which included all public health centres (n = 21) in the district, 1 out of 2 public hospitals, 2 out of 7 private hospitals, and 6 out of 22 private clinics. Facilities and hospitals were selected to be representative of three different epidemiological profiles: (1) endemic (located within Menoreh Hills), (2) non-endemic (outside Menoreh Hills) with only imported cases reported from 2017 to 2019, and 3) non-endemic (outside Menoreh Hills) with zero cases reported between 2017 and 2019 (*personal communication*). In Magelang, health systems interviews were conducted in a total of 32 health facilities, including 21 out of 29 public health centres, 1 public hospital, 2 out of 3 private hospitals, and 6 out of 20 private clinics.

The questionnaire was designed to align with national malaria elimination audits conducted by the National Committee of Malaria Elimination Assessment, which consists of experts from the Indonesian Ministry of Health, and external partners from universities, WHO, UNICEF, professional organizations, malaria experts, NGOs, faith-based organizations (FBOs), Armed Force Indonesia, National Police of Republic Indonesia (POLRI), and other relevant stakeholders [6]. Questions were grouped into the following categories: facility catchment population and health seeking behaviour, quality and availability of microscopy testing materials, quality and availability of RDT testing materials, reference laboratory procedures, quality of microscopy and/or RDT training, training and supervision for case management, quality of case reporting (detailed list of questions are reported in Table 1). For 46 health facilities, longitudinal data of monthly malaria cases, routinely collected through the passive cases detection system, were collated from January 2017 to December 2019. This dataset included the number of health facility attendees, estimated facility catchment population, and the number of individuals tested for and diagnosed with malaria.

**Table 1:** Survey questions at health facilities in Kulon Progo and Magelang Districts

**Facility catchment**	
*catchment_population*	What is the estimated catchment population size of your health facility?
*monthly_outpatient*	What is the estimated average monthly number of outpatient attendees for your health facility?
*outside_catchment_percent*	What percentage of patients reporting at your facility are from outside your catchment area?
*atdistrict_outside*	What district are they normally a resident of?
**Health Seeking**	
*fever_percent*	What percentage of your total facility attendance reported fever in the last week?
*uncomp_mal_percent*	What percentage of attendees were diagnosed for uncomplicated malaria in the last year?
*severe_mal_percent*	What percentage of attendees were diagnosed for severe malaria in the last year?
**Microscopy materials**	
*microscopy_provided*	Does your facility provide malaria microscopy testing?
*functioning_microscope*	Do you have functioning electronic microscopes with dual eyepieces available?
*functioning_countingmeters*	Do you have functioning microscopy counting meters available?
*microscopy_stockout*	Has your facility experienced stock-outs of material to conduct microscopy in the last year?
*microscopy_stockout_month*	Which month was the stock-out?
*microscopy_stockout_length*	What was the length of the stock-out?
**RDT materials**	
*rdt_provided*	Does your facility provide malaria testing by RDT?
*rdt_stockout*	Has your facility experienced RDT stock-outs of material in the last year?
*rdt_stockout_month*	Which month was the stock-out?
*rdt_stockout_length*	What was the length of the stock-out?
**Antimalarial materials**	
*antimal_provided*	Does your facility provide antimalarials? (first-line ACT, injectable artesunate for severe malaria, or other)
*antimal_stockout*	Has your facility experienced stock-outs of antimalarials in the last year?
*antimal_stockout_month*	Which month was the stock-out?
*antimal_stockout_length*	What was the length of the stock-out?
**Reference laboratories**	
*reflab_confirm*	Do you confirm suspected samples with testing at reference laboratories?
*reflab_samples*	Which samples do you send to confirm at reference laboratories?
*reflab_criteria*	Specify criteria for sending samples to reference laboratory:
*Reflab*	Which reference laboratory do you send your samples to for confirmation?
**Microscopy training**	
*microscopy_staff*	Do you have staff available at your facility to conduct malaria microscopy?
*microscopy_training*	Have your staff received training on malaria diagnosis by microcopy?
*microscopy_training_date*	When was the last microscopy training received?
*microscopy_competency*	Was a competency certificate received?
**RDT training**	
*rdt_staff*	Do you have staff available at your facility to conduct RDTs?
*rdt_hf_training*	Have your staff received training on malaria diagnosis by RDT?
*rdt_hf_training_date*	When was the last training received?
*rdt_chw_training*	Have community health workers that report to your facility received training on malaria diagnosis by RDT?
*rdt_chw_training_date*	When was the last training received?
**Case training**	
*case_training*	Have your staff ever received training or attended workshops on malaria case management?
*case_training_date*	When was the last training received/workshop attended?
*treatment_sops*	Is a copy of the national malaria treatment guidelines or standard operating procedures on malaria case management available in your facility?
*Supervision*	Has your facility received supervisory visits from a district health officer or consultant in the last year?
*supervision_date*	When was the last visit?
**Case reporting**	
*suspected_record*	Can you show me the facility records for number of patients suspected for malaria in the last 2–3 years?
*no_record_suspected_reason*	What are the reasons for not keeping records on patients suspected?
*tested_record*	Can you show me the facility records for number of patients tested for malaria in the last 2–3 years?
*no_record_tested_reason*	What are the reasons for not keeping records on patients tested?
*cases_record*	Can you show me the facility records for number of confirmed malaria cases in the last 2–3 years?
*no_record_cases_record*	What are the reasons for not keeping records on confirmed cases?
*vivax_record*	Can you show me the facility records for number of confirmed *P. vivax* cases in the last 2–3 years?
*no_record_vivax_reason*	What are the reasons for not keeping records on confirmed *P. vivax* cases?
*database_record*	Can you show me the online record of your facility’s submissions on the number of patients suspected, tested and confirmed for malaria to the national database in the last 2–3 years?
*test_case_definition*	What is your case definition/criteria to test a patient for malaria?
*vivax_definition*	What is the case definition for a *P. vivax* relapse (as opposed to a case) for this facility?
*vivax_investigation*	Is this facility able to conduct epidemiological investigations to confirm *P. vivax* relapse?
*imported_definition*	What is the case definition for an imported case for this facility?
*imported_investigation*	Describe the epidemiological investigation process your district uses to confirm if a case is imported or indegenous

### Data analysis

SSe is traditionally estimated via a scenario tree modelling and is given by the product of the tree branches representing the flow of an infected individual through the system (based on detection of malaria symptoms) [13]. We developed an adapted version of the scenario tree model, embedded in a statistical framework to estimate model parameters using both routinely collected PCD data and interview data collected through the health facility.

**Fig. 2:**
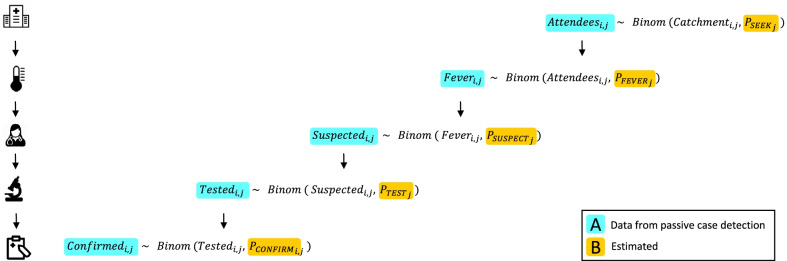
Schematic representation of the surveillance data flow for estimating the sensitivity of a surveillance system, for the ith month and *j*th health facility. ${Attendees}_{i,j}$: patients attending the health facility, ${Fever}_{i,j}$: patients reporting fever, ${Suspected}_{i,j}$: patients being suspected for malaria, ${Tested}_{i,j}$: patients being tested for malaria, ${Confirmed}_{i,j}$: patients being confirmed with malaria, ${{P}_{SEEK}}_{j}$: probability of care seeking, ${{P}_{FEVER}}_{j}$: probability of having clinical symptoms (fever), ${{P}_{SUSPECT}}_{j}$: probability of being suspected for malaria, ${{P}_{TEST}}_{j}$: probability of being tested for malaria, ${{P}_{CONFIRM}}_{i,j}$: probability of being confirmed with malaria

The statistical framework consists of the following elements. Consider $J$ health facilities and a passive case detection longitudinal series of $I$ surveillance months. For the *i*th month and *j*th health centre, the following data can be collected: number of patients attending the facility (${Attendees}_{i,j}$), number of people reporting fever (${Fever}_{i,j}$), number of people suspected for malaria (${Suspected}_{i,j}$), number of people tested for malaria (${Tested}_{i,j}$), and number of people with confirmed malaria (${Confirmed}_{i,j}$). Each of these quantities can be considered as a conditional proportion of the previous parameter, and the entire flow of data can therefore be described as a series of binomial processes (Fig. 2). Probabilities, and their uncertainty, are assigned at each branch point, estimated from either empirical data or expert-opinion [14, 15].

Note that the number of suspected cases can be higher than the number of patients with fever as other symptoms may prompt recommendation for malaria diagnosis. In such a case, a Poisson process can be used instead, i.e., ${Suspected}_{i,j}\sim Poisson({Fever}_{i,j} \times {{ P}_{SUSPECT}}_{j})$.

Most of these empirical data (Fig. 2A) can be directly observed with routinely collected data (${X}_{j,k}$), whereas the probabilities driving this cascade (Fig. 2B) can be estimated as:

1 $$logit \left({P}_{j}\right)= \alpha + {\sum }_{k=1}^{n}{\beta }_{k}{X}_{j,k}$$

Equation (1) is a logistic regression, where ${P}_{j}$ is the probability being estimated, and the linear predictor on the right-hand-side of the expression comprises a set of n coefficients $\beta$ and n explanatory variables X collected at ${j}^{th}$ health centre via the survey questionnaires.

Here, we focus on two branches of the cascade which can be estimated with survey-collected data: ${P}_{SEEK}$ and ${P}_{TEST}.$

For each *i*th month and each *j*th health facility, we considered the number of ${Attendees}_{i,j}$, as reported by the PCD system. This data is described as

2 $${Attendees}_{i,j} \sim Binom\left({Catchment}_{i,j}, {{P}_{SEEK}}_{j}\right)$$

where the likelihood of attending the *j*th health centre, given the monthly catchment population ${Catchment}_{i,j}$, is determined by the probability of care seeking ${{P}_{SEEK}}_{j}$.

Similarly, we considered the number of people tested for malaria in each *i*th month, at each *j*th health facility, ${Tested}_{i,j}$, as a process where the number of people tested is assumed to be binomially distributed according to

3 $${Tested}_{i,j} \sim Binom\left({Fever}_{i,j}, {{P}_{TEST}}_{j}\right)$$

Note that in this case study there was no empirical data for ${Suspected}_{i,j}$ available, hence we considered the number of patients with fever symptoms to be a proxy of those suspected for malaria.

To define both the two sets of probabilities ${{P}_{SEEK}}_{j}$ and ${{P}_{TEST}}_{j}$ they were modelled using Eq. (1). In both models, we allowed for variation at the district-level, included in the model as a random intercept by district.

To model ${{P}_{SEEK}}_{j}$, explanatory variables included whether the health centre provides antimalarial drugs ($antimal\_provided$), whether the health centre experienced a recent stockout of antimalarial drugs ($antimal\_stockout$) and the length in months of the last stock-out ($antimal\_stockout\_length$). In addition, the probability of care seeking is known to be affected by the accessibility to the health centre [16]. Therefore, we calculated the average travel time ($t\_time$) to each health centre following the approach proposed by Weiss et al. 2015 [17], defined the effective catchment areas of each facility as suggested by Nelli et al. 2020 [16], and used the average travel time in each catchment area as further covariate.

To model ${{P}_{TEST}}_{j}$, explanatory variables included whether the health centre had functioning microscopy equipment ($microscopy\_function$), whether functioning counting meters were available ($microscopy\_meters$), whether there was a recent stockout of microscopy materials ($microscopy\_stockout$), whether the health centre had staff available to conduct microscopy testing ($microscopy\_staff$), whether staff had undergone recent microscopy training ($microscopy\_training$), whether RDT testing was a service available at the health facility ($rdt\_provided$), whether staff was available to conduct RDT testing ($rdt\_staff$), whether there was a recent stockout of RDT materials ($rdt\_stockout$), whether a copy of the national malaria treatment guidelines or standard operating procedures on malaria case management were available for staff in the facility ($treatment\_sops$), and whether the facility received supervisory visits from a district health officer or consultant in the last year ($supervision$).

All the dichotomous variables were treated as binary variables (coded as 1 if “Yes”, 0 if “No”). The analysis was conducted using Bayesian model fitting with the program *JAGS* [18], interfaced with the statistical environment *R* [19] via the package *rjags* [20]. We used Markov Chain Monte Carlo (MCMC) algorithms to fit the two models. Non-informative normally distributed priors with a mean of zero, corresponding to a null hypothesis of no-effect for each covariate, where chosen for all $\beta$ coefficients.

## Results

Antimalarial drugs were reported being available at 0.32 of the facilities. Of these, 0.25 had experienced a recent stock-out of antimalarial drugs, lasting on average 3.6 months (range 1–8) (Table 2). When modelling the probability of attending the health facilities (Table 3), we found that clinics that regularly provide antimalarial drugs are 3 times more likely to be attended for care-seeking ${P}_{SEEK}$ (Odds Ratio [OR] = 2.98, 95% Credible Intervals [CI]: 1.54–3.16). In addition, we found that clinics that experienced antimalarial drug stock-out are less likely to be attended for care-seeking (OR = 0.31, CI: 0.30–0.39). We also found a negative effect of the average travel time in the catchment area, indicating that accessibility plays a major role in the probability of care-seeking. In particular, for every hour of travel time, we might expect a decrease of probability of care seeking of approximately 0.04. No differences in ${P}_{SEEK}$ between the two districts were found.

**Table 2:** Summary of health facility responses in Kulon Progo and Magelang Districts

Question	Answer (%)		
Antimalarial materials	Yes		No
* antimal_provided*	32.3		67.7
* antimal_stockout*	Yes	No	
	25.0	55.0	
* antimal_stockout_length* (months /range)	3.6/1–8		
Microscopy materials	Yes		No
* microscopy_provided*	82.3		17.7
* functioning_microscope*	Yes	No	
	98.0	2.0	
* functioning_countingmeters*	48.0	52.0	
* microscopy_stockout*	13.7	86.3	
* microscopy_stockout_length*	3.6/1–12		
RDT materials	Yes		No
* rdt_provided*	14.5		85.5
* rdt_stockout*	Yes	No	
	44.4	55.6	
* rdt_stockout_length* (months/range)	4.0/1–6		
Reference laboratories	Yes		No
* reflab_confirm*	64.5		35.5
* reflab_samples*	Yes	No	
	53.8	46.2	
Microscopy training	Yes		No
* microscopy_staff*	79.0		21.0
* microscopy_training*	Yes	No	
	89.8	10.2	
* microscopy_competency*	56.8	43.2	
* microscopy_training_length (years since last /range)*	2.9/0.9–16.9		
RDT training	Yes		No
* rdt_staff*	35.5		64.5
* rdt_hf_training*	Yes	No	52.4
	47.6	52.4	
* rdt_hf_training_length*	3.9/1.8–7.9		
	Yes		No
* rdt_chw_training*	30.6		69.4
* rdt_chw_training_length (years since last /range)*	1.5/0.9–2.0		
Case training	Yes		No
* case_training*	50.0		50.0
* case_training_length (years since last /range)*	3.5/0.9–17.0		
* treatment_sops*	61.3		38.7
* supervision*	30.6		69.4
* supervision_lenght (years since last /range)*	1.2/0.9–4.9		
Case reporting	Yes		No
* suspected_record*	56.5		43.5
* tested_record*	56.5		43.5
* cases_record*	51.6		48.4
* vivax_record*	51.6		48.4
* database_record*	32.3		67.7

Responses to each survey question are summarised by proportion of total facilities interviewed (or average and range, in case of numerical values). Not shown in the summary table are descriptions of each facility’s malaria case definition, case and relapse definition for *P. vivax*, and *P. vivax* case investigation procedure

**Table 3:** Result of Bayesian models of probability of care seeking (${P}_{SEEK}$) and probability of being tested for malaria (${P}_{TEST}$), as a function of antimalarial availability at health facilities in Kulon Progo and Magelang Districts

Model	Variable	*β*	95% LCI	95% UCI
*P*_*SEEK*_	*(intercept)*	− 3.501	− 3.589	− 2.640
	*antimal_provided*	1.093	0.430	1.151
	*antimal_stockout*	− 1.164	− 1.195	− 0.921
	*travel_time*	− 0.044	− 0.075	− 0.041
*P*_*TEST*_	*(intercept)*	− 6.100	− 6.941	− 5.454
	*microscopy_function*	1.992	1.290	3.026
	*microscopy_meters*	0.549	0.532	0.566
	*microscopy_staff*	3.095	2.717	3.482
	*microscopy_stockout*	− 1.098	− 1.145	− 1.001
	*rdt_provided*	0.052	− 0.450	0.314
	*rdt_staff*	0.015	0.009	0.032
	*rdt_stockout*	− 0.625	− 4.659	4.593
	*supervision*	0.060	0.037	0.085
	*treatment_sops*	0.110	0.088	0.131

*β*: mean of posterior distribution, LCI: lower credible interval, UCI: upper credible interval

Microscopy was available in 0.82 of the health facilities (Table 2). Of these, 0.98 had functioning equipment at the time of the interview, but only 0.48 had available counting meters. Stock-out of microscopy material was experienced by only 0.14 of the health facilities, with an average length of 3.6 months (range 1–12). In terms of availability and training of staff in microscope usage, 0.79 of the health facilities had staff trained to conduct microscopy analysis at the time of the interview. Of these, 0.90 had received training on malaria diagnosis by microscopy, on average 2.9 years before the interview data (range 0.9–16.9) (Table 2).

According to national guidelines, RDT-based diagnosis is used by facilities that do not have microscopy services available. Malaria diagnosis via RDT was provided by only 0.15 of the health facilities, and 0.44 of them had experienced recent stock-out of RDT materials, for an average of 1.5 months (range 0.9–2.0). Staff who were able to conduct RDT testing were however available in 0.36 of the health facilities offering RDT testing services, with 0.45 of them having received recent training, on average 3.9 years prior to the interview date (range 1.8–7.9). In addition, community health workers reporting to health facilities received training on malaria diagnosis by RDT in 0.31 of cases, on average 1.5 years prior to the interview date (range 0.9–7.9) (Table 2).

Staff in 0.50 of the health facilities had either received training or attended workshops on malaria case management, on average 3.5 years prior to the interview (range 0.9–17.0). Copies of the national malaria treatment guidelines or standard operating procedures on malaria case management were available in 0.61 of the facilities. 0.30 of the facilities received supervisory visits from a district health officer or consultant, on average 1.2 years prior to the interview date (range 0.9–4.9) (Table 2).

In terms of case reporting, 0.57 of the health facilities presented the official records for the number of patients suspected and tested for malaria in the last 2–3 years. 0.52 of the facilities were able to show records for the number of confirmed malaria cases in the last 2–3 years (for both *P. vivax* as well), but only 0.32 of them were able to show the online record of the facility's submissions on the number of patients suspected, tested and confirmed for malaria to the national database (Table 2).

The variables related to microscopy diagnosis were those with the stronger effect on the probability of being tested for malaria, as they showed the higher absolute values in the coefficient of ${P}_{TEST}$ (Table 3). In particular, and unsurprisingly, higher probability of test was associated with the availability of staff to conduct microscopy diagnosis (OR = 22.09, CI: 15.14–32.52), followed by the availability of functioning microscopy equipment at the time of the interview (OR = 7.33, CI: 3.63–20.61). Having experience recent stockout of microscopy material was associated with a lower probability of testing (OR = 0.33, CI: 0.32–0.37). In addition, having a copy of the national standard operating procedures available in the health facility, and having received regular supervisory visits from a district health officer, increased ${P}_{TEST}$, although with a smaller effect relative to the availability of staff and functioning equipment (OR 1.12 and 1.06 respectively). The variables related to RDT equipment and training did not show a clear effect on ${P}_{TEST}$. No differences in ${P}_{TEST}$ between the two districts were found.

**Fig. 3:**
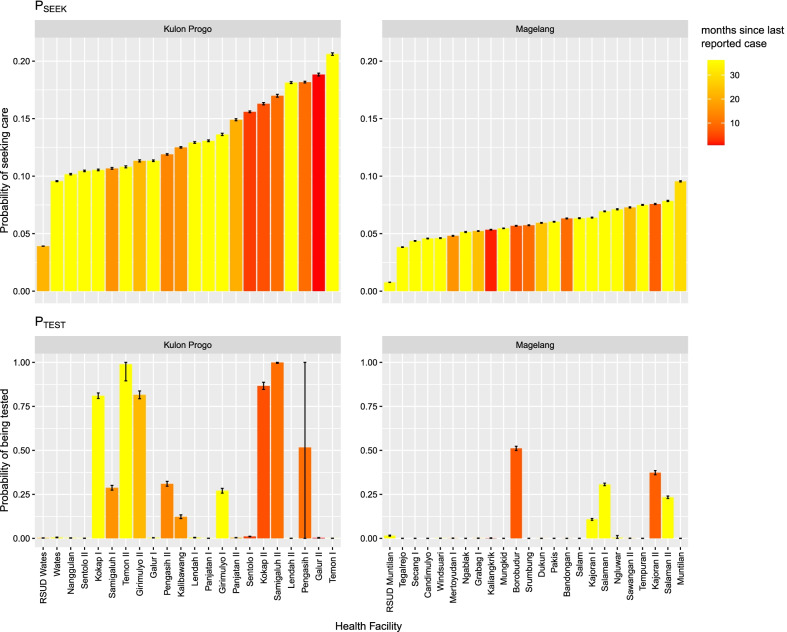
Expected probability of care seeking (P_SEEK_) and probability of being tested for malaria (P_TEST_), obtained from the mean of the posterior distribution of the Bayesian model fit, together with their standard deviation (error bars)

**Fig. 4:**
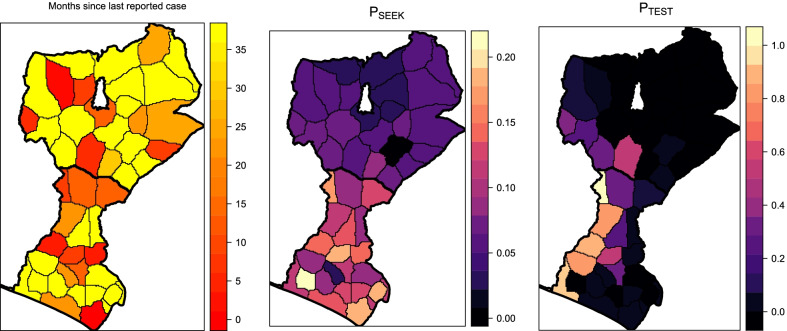
Months since last reported malaria case, expected probability of care seeking (P_SEEK_) and probability of being tested for malaria (P_TEST_), obtained from the mean of the posterior distribution of the Bayesian models, in Magelang and Kulon Progo districts (Indonesia)

**Fig. 5:**
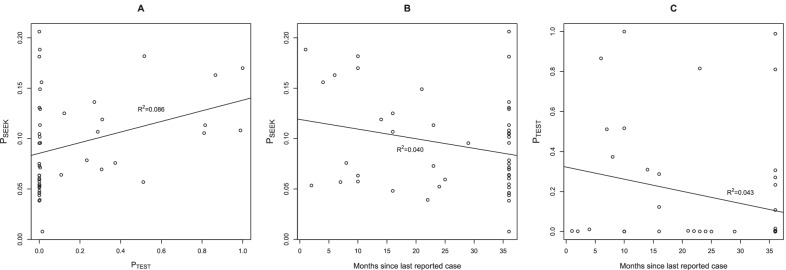
Relationship between **A** probability of care seeking (P_SEEK_) and probability of being tested for malaria (P_TEST_), **B** ${P}_{SEEK}$ and time since last reported malaria case, **C** ${P}_{TEST}$ and time since the last reported malaria case, obtained from the mean of the posterior distribution of the Bayesian models, in Magelang and Kulon Progo districts (Indonesia)

Figures 3 and 4 show the expected probability of care seeking and probability of being tested for malaria, obtained from the mean of the posterior distribution of the Bayesian models. A moderate level of heterogeneity between facilities/catchment areas can be noted in particular for the estimate of the probability of monthly seeking behaviour, with values ranging from 0.01 to 0.21 (mean ± sd = 0.09 ± 0.05). There was greater heterogeneity in the probability of being tested given the number of patients presenting fever symptoms, with a facility-level mean posterior distribution of ${P}_{TEST}$ ranging between 0.00 and 1.00 (mean ± sd = 0.16 ± 0.29), indicating a wide variability between facilities. Notably, ${P}_{SEEK}$ and ${P}_{TEST}$ did not appear to be strongly correlated, i.e. facilities in communities with a high probability of seeking care does not necessarily imply a high probability of being tested for malaria at the facility (Fig. 5A). In addition, we assessed whether ${P}_{SEEK}$ and ${P}_{TEST}$ were associated with time since the last malaria case was reported (in December 2019) to determine if any health system surveillance functions become less stringent when no cases are reported for a longer period of time, but none found to be correlated (Fig. 5B and C).

## Discussion

We presented a statistical framework to characterise and assess malaria surveillance systems using empirical data collected via health facility surveys and a series of modelled relationships between key health systems parameters. The framework was assessed in near elimination settings in two districts on Java, Indonesia.

Overall, the probability of care seeking by the local catchment population was most dependent on availability of antimalarial drugs, absence of antimalarial stockouts, and average travel time to health facilities. In this setting, each additional hour of travel time was associated with a 4% decrease in probability of care seeking. This further supports the important role that Community Health Workers (CHWs) have in not only providing easy access to testing and treatment but also for improving the sensitivity of malaria surveillance. In regions where access to health facilities is particularly low, passive case detection may need to be supplemented with active case detection to detect any potential infections in the community. To guide programmatic implementation of surveillance strategies, future analysis can seek to determine a threshold in average travel time above which care seeking behaviour is dramatically impacted [16], so that active surveillance can be targeted in these areas or provision made for additional facilities. Other factors can affect care seeking that we could not capture in this model without additional empirical data, such as the availability of health insurance or facility tier. On average, citizens in Kulon Progo and Magelang have good insurance coverage (*personal communication*) so it is unlikely to be an important factor in this setting. However, the heterogeneity of health insurance coverage across the country may significantly affect the care seeking in other settings in Indonesia [21]. In addition, other sociodemographic factors are known to encourage or impede self-reporting, such as poverty and education [22], ethnicity [23, 24] and language barriers [25]. Cultural beliefs and self-medication may widely vary across Indonesia and can affect the care seeking as well [26]. If available and quantifiable at the facility level, these additional variables can easily be included in the model framework.

Microscopy is the national standard for malaria diagnosis in Indonesia due to the circulation of multiple malaria species, and capacity overall was generally high, with microscopy available in over 80% of facilities. Of these, 98% had functioning microscopy equipment and 90% had received staff training in microscopy-based diagnosis. The probability of testing for malaria was most dependent on the availability of staff and functioning microscopy equipment, as well as recency of receiving staff training in microscopy.

Although here we focussed on a cascade that captures the flow of an infected individual through the system from the development of symptoms, we are aware that the evaluation of the sensitivity of the malaria surveillance system would not be complete without accounting for subclinical malaria infection. Estimating the proportion of asymptomatic cases in a population is however challenging and related to many factors including the degree of acquired immunity, care seeking behaviours, amongst others [27]. Availability of active case detection data would “boost” our model and improve estimates. As the additional data to estimate such proportion was not available our proposed approach was made purposely flexible to inform specific components and account for the added uncertainty. For example, the probability of having clinical symptoms,${P}_{FEVER}$, can be informed by prior knowledge of symptomatic cases in malaria (proportions and standard deviation), from previous studies and/or published literature.

In addition, here ${P}_{FEVER}$ is used as a proxy for probability of having clinical malaria symptoms. This is not an ideal proxy and can lead to an over- or under-estimate of ${P}_{TEST}$ depending on the probability of non-malarial febrile illnesses in the population [28]. Ideally, differentiating between malaria and non-malarial fevers would improve the model fit as this bias is expected to affect the precision and interpretation of both ${P}_{SEEK}$, and ${P}_{FEVER}$. If information on the number of non-malarial febrile cases reporting to the facilities per month or the number of the febrile patients that are suspected of malaria based on the local clinical algorithm are known, this can be accounted for in the model. Unfortunately, this data but was not available as part of this case study and this limitation is acknowledged. However, the model presented here provides a flexible framework to incorporate these data and crucially, this research highlights the key information that could be collected to improve SSe estimation.

The probability of being suspected of malaria, when not recorded by health facilities, could also be estimated based on the probability of being tested, assuming that clinicians only test those they suspect of having malaria. Alternatively, if prior knowledge on the proportion of malaria attributable fevers is available, this can be applied and inform more specific priors. In addition, other symptoms that are considered in the national recommendation for diagnosis algorithm for clinical malaria can be used to infer a more accurate estimate of suspected cases. Future variations of this framework can aim to explore this in more depth and collect data on suspected cases and/or the clinical indicators other than fever used to screen for potential cases as well as test the ideal approach where the data is not routinely collected. Given its role in the model, recording the number of people suspected of malaria may be one aspect where surveillance could be strengthened for countries approaching elimination, but methods to more accurately assess adherence to malaria diagnosis guidelines will need to be considered.

The parameters that we used to estimate the probabilities represent a “snapshot” of the health facilities’ characteristics at a given time point, but the routine surveillance data informing the models are a temporal series collected three years preceding the interview date. The lack of temporal congruence could be the reason why there were so few factors associated with ${P}_{TEST}$ and ${P}_{SEEK}$ based on the data available. Future research is required to identify the optimal frequency and operational methods to obtain this data as well as the appropriate model framework to take this temporal (including seasonality) and any spatial dimensions into account.

*P. falciparum*, *P. vivax*, and other species have unique challenges in elimination and surveillance [29]. Unfortunately, for this specific case study, detailed monthly data on different parasites was not available. Future applications of our model will need to account for different *Plasmodia* species, for example estimating different probabilities of being suspected depending on the geographical area or season, the different RDT sensitivities associated with the different species, and, in the case of P. vivax, accounting for a relapse compared to incident infection.

Our modelling framework focuses primarily on data that can be captured at health facility level as this will be the primary data source of certification of elimination it doesn’t explicitly address the role of Community Health Workers (CHWs). Test results collected by CHWs in Indonesia are typically reported to the health facilities where they are assigned (and responsible for supervision). Data captured by CHWs are, therefore, an extension of health facility surveillance. However, future application of our framework can look at the added value of including data from CHWs, in terms, for example, of their role in the estimation of ${P}_{TEST}$ and ${P}_{SEEK}$. To assess this directly, would require data modified questionnaires at health facility and/or direct interviews to CHWs.

This study provides a model framework to assess health systems surveillance capacity for malaria elimination, using a combination of data routinely recorded in health facilities and data obtained through simple-to-administer health facility surveys. Some challenges faced when conducting the health facility surveys include potential limitations in manually recorded private health facility data, which (unlike public facilities) are not mandated to conduct routine malaria surveillance and control activities such as recording/reporting of or response to malaria cases. Overall, however, we were able to collect a higher resolution of data than district-level surveys conducted through the Indonesian national audit. This builds the evidence to inform the design of standardised surveys to assess health systems readiness for malaria elimination surveillance, using a statistical approach to weight the importance of variables collected and provide a basis for programmatic prioritization. Further validation of this model framework in other areas, at all levels of endemicity, will allow determination of the degree to which survey questions or model variables need to be tailored to local settings and health systems (e.g., national guidelines for diagnostics used in malaria confirmation, existing national audit process, clinical diagnosis guidelines).

## Conclusions

Our framework provides a data-driven method to estimate the effectiveness of a health system to detect malaria, quantifying key steps in the continuum from care seeking to facility-confirmed malaria diagnosis and reporting. Our results allowed identification of key factors associated with each parameter, which could be improved to strengthen the surveillance system at all levels of malaria transmission, enhancing robustness of data for public health decision-making.

## Data Availability

The anonymised data collected to support this analysis are available from the corresponding author upon reasonable request.

## Abbreviations

CHW: Community health worker
FBO: Faith-based organization
FFI: Freedom from infection
LSHTM: London School of Hygiene and Tropical Medicine
MCMC: Markov Chain Monte Carlo
NGO: Non-governmental organization
OR: Odds ratio
${P}_{SEEK}$: Probability of care seeking
${P}_{FEVER}$: Probability of having clinical symptoms
${P}_{SUSPECT}$: Probability of being suspected for malaria
${P}_{TEST}$: Probability of being tested for malaria
${P}_{CONFIRM}$: Probability of being confirmed with malaria
*P. falciparum*: *Plasmodium falciparum*
*P. vivax*: *Plasmodium vivax*
PCD: Passive case detection
POLRI: National Police of Republic Indonesia
RDT: Rapid diagnostic test
SSe: Surveillance system sensitivity
UGM: Universitas Gadjah Mada
UNICEF: United Nations Children’s Fund
WHO: World Health Organization
